# Speech target modulates speaking induced suppression in auditory cortex

**DOI:** 10.1186/1471-2202-10-58

**Published:** 2009-06-13

**Authors:** Maria I Ventura, Srikantan S Nagarajan, John F Houde

**Affiliations:** 1 Department of Radiology, University of California, San Francisco, San Francisco, CA, USA; 2 Department of Otolaryngology, University of California, San Francisco, San Francisco, CA, USA

## Abstract

**Background:**

Previous magnetoencephalography (MEG) studies have demonstrated speaking-induced suppression (SIS) in the auditory cortex during vocalization tasks wherein the M100 response to a subject's own speaking is reduced compared to the response when they hear playback of their speech.

**Results:**

The present MEG study investigated the effects of utterance rapidity and complexity on SIS: The greatest difference between speak and listen M100 amplitudes (i.e., most SIS) was found in the simple speech task. As the utterances became more rapid and complex, SIS was significantly reduced (*p *= 0.0003).

**Conclusion:**

These findings are highly consistent with our model of how auditory feedback is processed during speaking, where incoming feedback is compared with an efference-copy derived prediction of expected feedback. Thus, the results provide further insights about how speech motor output is controlled, as well as the computational role of auditory cortex in transforming auditory feedback.

## Background

The role of auditory feedback in speech production is a topic of longstanding interest that has been investigated via a number of methods, most recently in studies using functional neuroimaging methods. Previous studies using magnetoencephalography (MEG) have revealed a phenomenon called speaking-induced suppression (SIS): a reduced response in auditory cortex to self-produced speech, compared with its response to externally-produced speech. These studies examined the M100 response, also called the N100m response, which is the most significant peak in the magnetic response of cortex occurring approximately 100ms after the onset of an auditory stimulus[1], and found a dampened auditory M100 response to a person's own voice when speaking compared to conditions in which a person listens to recorded speech being played back to them [2-4]. Researchers have also found that when self-generated voice sounds were different from the expected sounds, auditory cortex response was maximal, but if the output during speech production matched the expected sound, cortical activity in the auditory cortex was suppressed [5]. Heinks-Maldonado, Nagarajan and Houde [6] proposed a precise forward model for speech production. They suggested that a forward model operates in the auditory system during speech production, which caused maximal suppression of the auditory cortical response to the incoming sounds that most closely match the speech sounds predicted by the model. Researchers have argued that precise auditory suppression during speech allows the auditory system to distinguish between internally and externally produced speech sounds [7,8].

**Figure 1:**
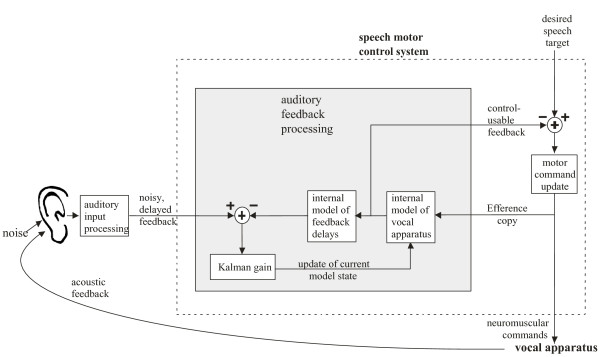
**The model of auditory feedback processing for speech proposed by Houde & Nagarajan (2007)**. It is based on an internal model of feedback generation that includes a model of the vocal apparatus and a model of feedback loop delays incurred by motor responses and sensory feedback processing. If the expected speech sound from the internal model closely matches the actual speech sound, the prediction error is small, which is seen as small activity in auditory cortex. However, if the actual speech sound does not match the prediction, there is a greater prediction error and thus greater activity in auditory cortex.

A working model of auditory feedback processing for speech that accounts for the SIS phenomenon has been proposed by J.F. Houde and S.S. Nagarajan [9](Figure 1). This model is a version of the Kalman filtering approach taken to model motor control in other domains [10-12]. A key part of this model is an internal model that represents the learned associations between vocal motor commands and their resulting acoustic sensory consequences, which we hypothesize is acquired when learning to speak (i.e. during babbling). During speaking, actual (noisy, delayed) incoming auditory feedback is compared with a feedback prediction derived from efference copy of the motor output commands, creating a feedback prediction error. It is this comparison process that we hypothesize is a principal cause of the speaking-induced suppression phenomenon seen in our MEG studies.

If Houde and Nagarajan's model of auditory feedback processing is correct, one would expect utterance rapidity and complexity to affect SIS because temporal misalignment between actual feedback and the prediction only affects the prediction error for dynamic articulations. Thus, the goal of this paper is to test the above two model predictions about differences in SIS for static versus rapid, dynamic speech targets. We did this by comparing differences between auditory processes during speech production compared to auditory processes during passive listening across three different conditions with various speech targets (/a/, /a-a-a/, /a - a-a - a/). If the model is correct, we would expect a maximal difference in magnitude of the M100 response between speak and listen tasks in the simplest condition (/a/). However, with increasing rate and complexity of utterances (/a-a-a/, /a - a-a - a/), the speaking induced suppression should be reduced, and the difference in magnitude of the M100 response between speak and listen amplitudes should be smaller in the complex utterances.

## Results

**Figure 2:**
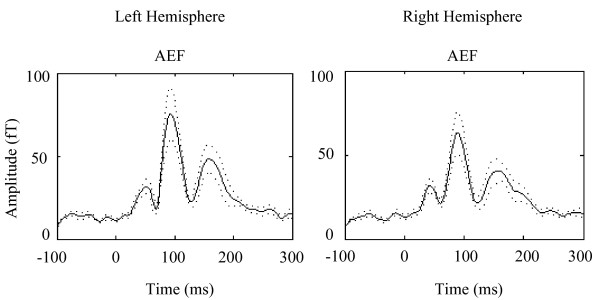
**The global field power (RMS) for the average Auditory Evoked Field**. AEF response was evoked with 120, 1 kHz pure tones. M100 response was observed in both hemispheres. There was no hemispheric difference for amplitude. However, there was a hemispheric difference for latency; mainly, the right hemisphere processed tones on average 9.5 msec faster than the left hemisphere. Standard Error (±) shown in dashed line.

We first examine the Auditory Evoked Field (AEF) response to simple tones in all subjects. The AEF M100 data revealed no hemispheric differences in the amplitude of the response from each hemisphere (Figure 2). However, there was a hemispheric difference for M100 latency, *p *= .002, showing that the right hemisphere processed pure tones more quickly than the left hemisphere across all 10 participants.

**Figure 3:**
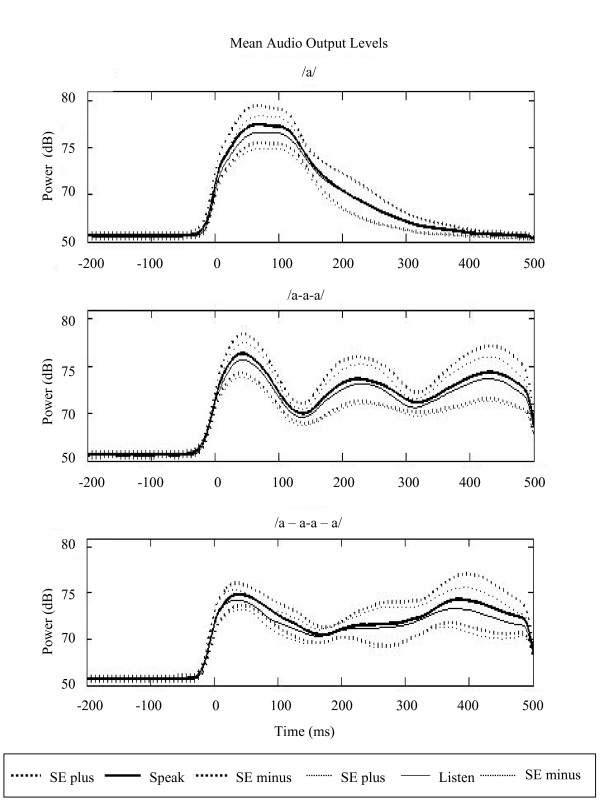
**Mean audio output levels across conditions**. The acoustic output data shows that participants produced /a/ more loudly than /a-a-a/ which they in turn produced more loudly than /a - a-a - a/. However, calibration of the acoustic stimuli ensured that the volume through the earphones was equivalent in both speak and listen tasks in each condition. Standard Error (±) shown in dashed line. Output shown as acoustic power in dB SPL (ambient baseline noise in the scanner room was 50 dB).

Second, we analyzed the acoustic output amplitude, i.e. volume through the earphones that subjects' heard, in both speak and listen tasks. This analysis revealed significant difference between conditions, *p *= 0.000033, but not across task. Overall, participants produced /a/ more loudly than /a-a-a/ which they in turn produced more loudly than /a - a-a - a/. However, no differences were found in the acoustic amplitude that the subject heard during the speak and listen tasks in these conditions. Responses to speech sounds during speaking and listening showed that M100 listen amplitudes decreased as the volume of the stimuli decreased (see Figure 3).

An analysis of the sensor Root Mean Square (RMS) M100 amplitude data during speech tasks revealed significant hemisphere and task differences. A repeated measures ANOVA with condition, task and hemispheres as factors revealed significant differences for hemisphere, *p *= .0336, and task, *p *= 0.000035. In contrast, M100 latency data revealed no significant differences.

Source space analysis using virtual sensors were used to analyze the M100 response arising from auditory cortex in each hemisphere in a 3 × 2 × 2 repeated measure ANOVA. The virtual sensor M100 amplitude data revealed significant differences for hemisphere, *p *= 4.42e-08, task, *p *= .0001 and a trend towards significance for the interaction between condition and task, *p *= .0504. To further investigate this interaction between task (speak or listen) and condition (static versus dynamic speech targets), we compared virtual M100 amplitude data from the simple speech target (condition 1) versus dynamic speech targets (conditions 2 and 3 combined). This ANOVA revealed significant differences for hemisphere, *p *= 0.000003, task, *p *= 0.0005, and a significant interaction between task and condition, *p *= .0353. Interestingly, the interaction between hemisphere and task did not reach significance, *p *= .0878 suggesting that task effects are similar across the two hemispheres.

**Figure 4:**
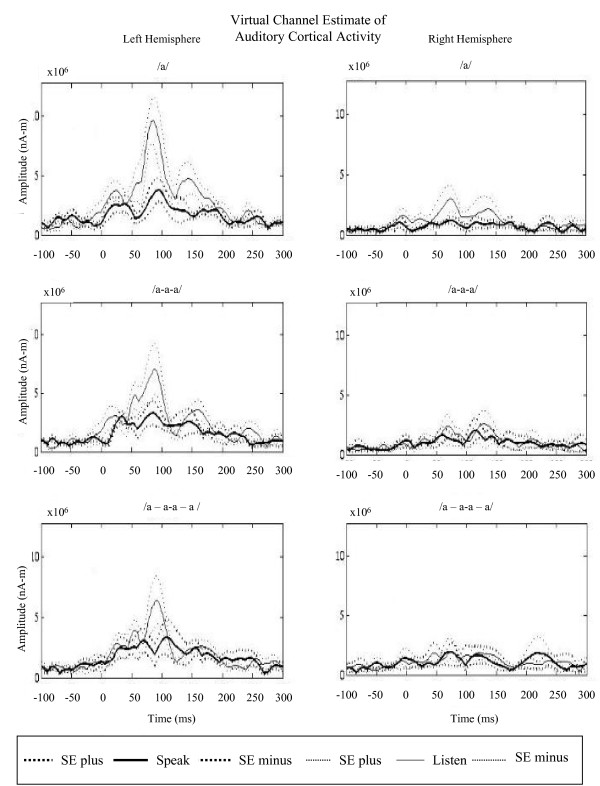
**Virtual sensor M100 amplitude of SIS in auditory cortex**. When the activity in the auditory cortex is isolated, a dampened response is observed in the right hemisphere. Maximal difference between speak and listen amplitudes was observed in the first condition with the simplest utterance, /a/. As the speech stimuli increased in rate and complexity, the difference between speak and listen amplitudes was reduced. Standard Error (±) shown in dashed line.

**Figure 5:**
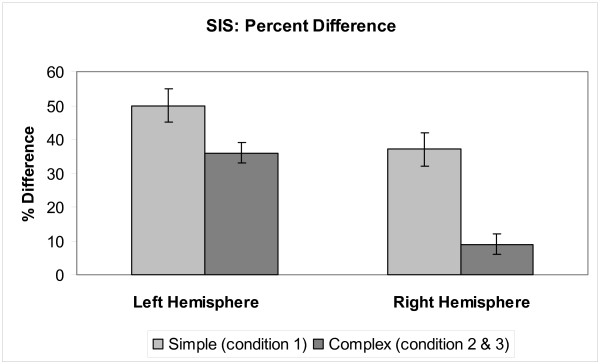
**Speaking Induced Suppression (SIS) percent difference: (amplitude_listen _- amplitude_speak_)/amplitude_listen_**. We compared SIS in simple (condition 1) vs. complex (conditions 2 & 3) across hemispheres.

Additional analyses of the virtual sensor amplitude data revealed differences in the responses between speak and listen tasks between conditions (see Figures 4 and 5). To specifically examine these differences across conditions independent of task, the percent difference was calculated per condition: (amplitude_listen _- amplitude_speak_)/amplitude_listen_. Results for the left hemisphere were as follows: in condition 1 (/a/), mean listen amplitude was 9.5 nA-m and mean speak amplitude was 4.0 nA-m, resulting in a percent difference between speak and listen amplitudes of 58%; in condition 2 (/a-a-a/), mean listen amplitude was 7.0 nA-m and mean speak amplitude was 3.5 nA-m, resulting in a percent difference of 50%; and in condition 3 (/a - a-a - a/), mean listen amplitude was 5.9 nA-m and mean speak amplitude was 3.8 nA-m, resulting in a percent difference of 35%. This analysis showed that the greatest difference between speak and listen amplitudes in the auditory cortex occurred in the simplest speaking condition. As the speech stimuli became more rapid and complex, the differences between speak and listen amplitudes decreased. A similar trend was also observed in the right hemisphere, although there was an overall dampened response to speech stimuli in the right hemisphere: in condition 1, mean listen amplitude was 3.5 nA-m and mean speak amplitude was 2.2 nA-m, resulting in a percent difference between speak and listen amplitudes of 38%; in condition 2, mean listen amplitude was 3.3 nA-m and mean speak amplitude was 2.8 nA-m, resulting in a percent difference of 15%; and in condition 3, mean listen amplitude was 2.35 nA-m and mean speak amplitude was 2.22 nA-m, resulting in a percent difference of 5%. The difference between speak and listen amplitudes was reduced as the utterances became more rapid and complex. To specifically test whether any increase in dynamics of speech target would increase SIS, we compared SIS percent differences from the simple speech target (condition 1) versus dynamic speech targets (conditions 2 and 3 combined) across hemispheres. This two-way ANOVA with condition and hemisphere as factors, revealed a significant difference for condition, *p *= 0.0003, but neither hemisphere, *p *= 0.8, nor the interaction between hemisphere and condition, *p *= 0.39, was significant. Therefore, SIS modulation for dynamic targets is bilateral, in spite of overall hemispheric differences in both speak and listen tasks.

No differences were observed in sensor RMS or virtual sensor M100 response latencies in the speech tasks.

## Discussion

Rapidity and complexity of the uttered syllable appears to modulate SIS of the M100 amplitude. SIS percent differences were largest with simple, static utterances in condition 1, smaller with rapid utterances in condition 2, and smallest with complex utterances in condition 3. Thus, the greatest difference between speak and listen M100 amplitudes was found in the static speech target (/a/), compared to the dynamic utterances (/a-a-a/ and /a - a-a - a/). These findings are consistent with predictions from our model of speech feedback processing. The greatest speaking induced suppression was observed in condition 1 with the simple utterance presumably because the internal representation, or mental model, for that utterance was largely static and therefore easy to produce and match. However, with increasing rate and complexity of utterances (conditions 2 & 3), the auditory feedback predictions became more dynamic and more difficult to keep in temporal registry with the incoming auditory feedback, resulting in a poorer match with it, and, thus, a less suppressed response.

The differences in amplitude results across conditions are also in accord with Houde and Nagarajan's (2007) model of speech feedback processing: one's expectation for a speech sound (including volume) is related to the activity observed in the auditory cortex. If a participant spoke /a/ loudly, that participant could predict the sound of that utterance and the auditory cortex will not be "surprised" by the volume of the utterance. In such a scenario, one would expect to observe attenuated activity, or reduced activity in the auditory cortex. If a participant spoke /a-a-a/ at a reduced volume, that participant could still predict the sound of that utterance and one would again expect to observe attenuated activity in the auditory cortex. However, during the listen task, participants could not predict the sound or volume of the auditory stimuli. Therefore, the auditory cortex behaved correspondingly: larger amplitudes were observed with louder stimuli, and smaller amplitudes were observed with more quiet stimuli. This is directly related to the prediction/expectancy aspect of the model proposed by Houde and Nagarajan: if one's internal representation for a speech sound (including volume) matches the actual speech sound, then suppression or attenuation of cortical activity in the auditory cortex is observed. This process of matching one's internal representation of a speech sound to the actual speech sound is only possible in the speak task. On the other hand, all stimuli are unexpected during the listen task, and thus response in the auditory cortex should behave solely according to the properties of the auditory stimuli (i.e. larger M100 amplitudes with louder stimuli, etc.).

Given that different auditory stimuli were used in this study, the hemispheric differences in observed responses are noteworthy. Several studies have reported no hemispheric differences when tones were used [13,14]. This is consistent with our findings: significant activations were observed in primary auditory cortex (mainly, in Heschl's gyri) in both hemispheres when pure tones were used [1]. In contrast, previous literature suggests that the left hemisphere is dominant during speech and language perception [2,4]. Therefore, a more dominant response was expected to occur in the left hemisphere, in other words a dampened response in the right hemisphere was expected, when speech stimuli were used. During both the speak and listen tasks, we observed this overall dampened M100 amplitude response in the right hemisphere. However, the effect of condition on SIS is the same across both hemispheres. Thus, in spite of the overall hemispheric differences in response to speech, the processing of auditory feedback during speaking may be similar across the two hemispheres.

## Conclusion

These findings provide additional support for our conceptual model of speech motor control, and as such provide the impetus to test other predictions from the model. In addition, these findings also provide better insights into the speech motor control system, and the computational role of auditory cortex in transforming auditory feedback. The SIS paradigm used in this study may benefit the study of disorders such as schizophrenia, in which patients lack the ability to distinguish between internally and externally produced speech sounds [15]. It may also benefit the study of speech production impediments such as stuttering [16,17], where altered auditory feedback has been shown to be fluency-enhancing.

## Methods

### Participants

Ten healthy right-handed English speaking volunteers (6 males, 4 females; mean age 25 years; range: 21–42) participated in this study. All participants gave their informed consent after procedures had been fully explained. The study was performed with the approval of the University of California, San Francisco Committee for Human Research.

### Experimental Design and Procedure

Calibration of the acoustic stimuli was conducted prior to starting the experiment to ensure that the volume through the earphones was equivalent in both speak and listen tasks. Each MEG session began and ended by recording Auditory Evoked Field (AEF) responses, which were elicited with 120 single 600-msec duration tones (1 kHz), presented binaurally at 70 dB sound pressure level (SPL).

**Figure 6:**
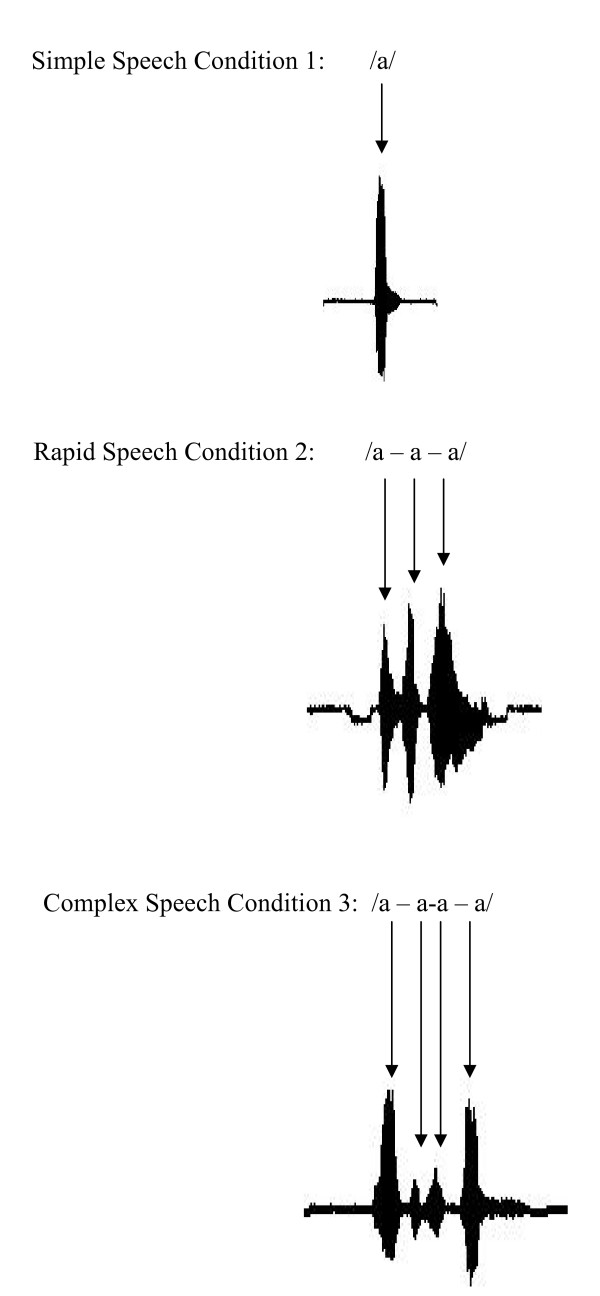
**A sample of the three speech targets**. In condition 1, participants were instructed to produce the utterance /a/ 75 times; average duration of each utterance /a/ was approximately 100 ms. In condition 2, participants were instructed to produce the utterance /a-a-a/ 75 times; approximately 50 ms separated each repetition of /a/. In condition 3, participants were instructed to produce the utterance /a - a-a - a/ 75 times; the rate of production of the two middle syllables was increased.

The experiment went as follows: participants viewed a projection monitor. The screen background was black, and three white dots appeared in the center of the screen. Each dot disappeared one by one to simulate a countdown (3-2-1). When all three dots disappeared and the screen was completely black, participants were instructed to speak the designated speech target. The experiment included three different speech targets (refer to Table 1 and Figure 6). In condition 1, also referred to as the simple speech condition, participants were instructed to produce the sound, /a/, 75 times (the speak task). The average duration of each utterance /a/ was approximately 100 ms. The 75 recorded utterances of /a/ were then played back to the participants (the listen task) in the same experimental design as the speak task: participants viewed the screen, "3-2-1" dots disappeared, and when the screen was black, the utterances were played back through the earphones unaltered. Conditions 2 and 3 are referred to as the rapid and complex speech conditions. These conditions had the same speak and listen tasks as in condition 1, except that in condition 2, participants were instructed to produce the sound, /a-a-a/, at a rapid consistent rate, with approximately 50 ms between each repetition of /a/. In condition 3, participants were instructed to produce a more complex sound, /a - a-a - a/, emphasizing the two middle syllables by changing the rate of production of the two middle syllables. During the speak and listen tasks, the MEG acquisition system recorded 3.5 seconds of data (i.e. 2 sec before the utterance and 1.5 sec after the utterance) at a sampling rate of 1200 Hz.

**Table 1:** The Experimental Design

**Simple Speech Condition 1: /a/**
**Speak **/a/ 75 times and record utterances.
**Listen **to playback of the 75 recorded /a/ utterances.
**Rapid Speech Condition 2: /a-a-a/**
**Speak **/a-a-a/ 75 times and record utterances.
**Listen **to playback of the 75 recorded /a-a-a/ utterances.

**Complex Speech Condition 3: /a - a-a - a/**
**Speak **/a - a-a -a/ 75 times and record utterances.
**Listen **to playback of the 75 recorded /a - a-a - a/ utterances.

A key feature of the experiment design related to analysis of the results is that the experiment is fundamentally a comparison between the speaking and listening conditions. Thus, although there are likely to be measureable differences in the audio recorded for productions of the three different speech targets used in the experiment (e.g., f0, formants), for each target, the audio heard by the subject is the same in both the speaking and listening conditions. Any response characteristics specific to the audio features of a given target are therefore removed when we compare responses to this target between the speaking and listening conditions.

A structural magnetic resonance image (MRI) was obtained for each participant at the Magnetic Resonance Science Center of UCSF. The whole head was imaged on a 1.5T General Electric scanner with approximately 124 slices, 1.5 mm thick.

### Data Acquisition and Processing

**Figure 7:**
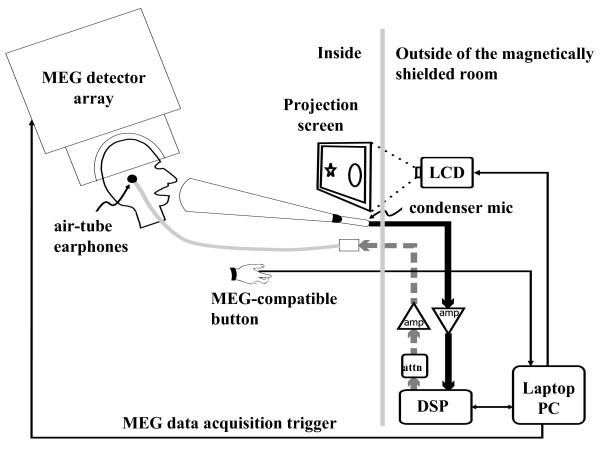
**The apparatus and experimental set up**. A 275 channel whole cortex MEG/EEG system was used (VSM MedTech Ltd.). All electronic equipment (the stimulus laptop, attenuator, digital signal processor, and amplifier) was placed outside of the magnetically shielded room. A directional microphone, plastic air tube earphones, an MEG compatible button, and monitor were in the room with the participant.

Magnetic fields were recorded using a 275 channel whole cortex MEG/EEG system (VSM MedTech Ltd., Coquitlam, British Columbia, Canada) from participants in a shielded room (Figure 7). Three fiducial points were defined on the surface of each participant's head using clear anatomic landmarks: left and right preauricular points and the nasion. The fiduciary points were used to superimpose the MEG data with the structural MRI for each participant [18].

Auditory Evoked Field (AEF) data (average response to 120 single pure tones) were band-pass filtered at 2–40 Hz, the third gradient of the magnetic field was calculated, and the DC offset was removed [6]. The average AEF was analyzed using equivalent current dipole (ECD) techniques [19]. Single dipole localizations for each hemisphere were obtained and the AEF response to 1 kHz pure tones elicited cortical activity in the auditory cortex in both hemispheres. Average MNI coordinates for left hemisphere (x, y, z) = -62.5, -20.6, 9.5 and for right hemisphere (x, y, z) = 61.8, -11.5, 8.18, revealed activation in primary auditory cortex (Brodmann areas 41, 42) and superior temporal gyrus in the normalized brain across subjects.

To assess activity changes in auditory cortex, two methods were used: standard Root Mean Square (RMS) averaging of detector measurements, as well as adaptive spatial filtering. Adaptive spatial filtering or beamforming is a spatial filtering technique that estimates the source signal specifically in the auditory cortex by attenuating uncorrelated activity in other brain regions, thereby increasing the signal to noise ratio [20-22]. The Synthetic Aperture Magnetometry (SAM) parameters were as follows: bandwidth 0–300 Hz, Z-threshold for weights = 5.0, and time windows from -200–300 ms. This results in a "virtual channel estimate" of the activation specifically localized in the auditory cortex during speech vocalizations. A virtual channel was created for each condition (/a/, /a-a-a/, /a - a-a - a/) and each task (speak or listen) per hemisphere (left or right) (Figure 4).

### Analysis

Statistical analysis was based on the M100 response, which was defined as the amplitude of the largest peak occurring within a designated time window, 60 to 120 ms post stimulus [1]. For the virtual channel estimate data, a three-way repeated measures Analysis of Variance (ANOVA) was conducted, and a separate ANOVA was conducted using the RMS data for comparison. A simple one-way, within subjects ANOVA was used to analyze the AEF responses to pure tones (amplitude and latency) in both hemispheres. One participant's data was excluded from RMS and virtual channel analyses due to severe contamination from dental artifacts during the speaking task; however, AEF analysis included all ten participants.

For static articulations, after speech onset, the effect of temporal misalignment errors between actual auditory feedback and the prediction is minimal because the articulators are moving slowly and feedback prediction is not changing quickly over time. Therefore, in the case of simple articulations, such as a single vowel /a/ in condition 1, temporal inaccuracies should have little effect on prediction error, and thus increase SIS. In contrast, for dynamic articulations, such as /a-a/, immediately after onset, the articulators are already in motion to realize the next articulatory goal (in this case, the glottal stop between the first and second productions of /a/). Any temporal misalignment between auditory feedback and the prediction will contribute to a larger prediction error since the feedback prediction is changing rapidly over time. Therefore, in the case of rapid, dynamic articulations in conditions 2 & 3, temporal inaccuracies should increase prediction errors, and thus decrease SIS.

Analysis of the acoustic output amplitude was conducted in order to verify that the volume participants heard through the earphones in both speak and listen tasks was equivalent. Peak amplitudes of the first syllable in all three conditions were analyzed using a one-way within subjects ANOVA.

## Authors' contributions

SSN and JFH conceived of the study and participated in its design. MIV coordinated the study, recruited participants and collected the imaging data. JFH and MIV performed statistical analysis. All authors contributed to writing the manuscript and approved the final draft.

## References

[B1] Reite M, Adams M, Simon J, Teale P, Sheeder J, Richardson D, Grabbe R (1994). Auditory M100 component 1: relationship to Heschl's gyri. Cognitive Brain Research.

[B2] Curio G, Neuloh G, Numminen J, Jousmaki V, Hari R (2000). Speaking modifies voice-evoked activity in the human auditory cortex. Human Brain Mapping.

[B3] Gunji A, Hoshiyama M, Kakigi R (2001). Auditory response following vocalization: a magnetoencephalographic study. Clinical Neurophysiology.

[B4] Houde JF, Nagarajan SS, Sekihara K, Merzenich MM (2002). Modulation of the auditory cortex during speech: an MEG study. Journal of Cognitive Neuroscience.

[B5] Hirano S, Kojima H, Naito Y, Honjo I, Kamoto Y, Okazawa H, Ishizu K, Yonekura Y, Nagahama Y, Fukuyama H, Konishi J (1997). Cortical processing mechanism for vocalization with auditory verbal feedback. NeuroReport.

[B6] Heinks-Maldonado TH, Nagarajan SS, Houde JF (2006). Magnetoencephalographic evidence for a precise forward model in speech production. NeuroReport.

[B7] Heinks-Maldonado TH, Mathalon DH, Gray M, Ford JM (2005). Fine-tuning of auditory cortex during speech production. Psychophysiology.

[B8] Eliades SJ, Wang X (2005). Dynamics of auditory-vocal interaction in monkey auditory cortex. Cerebral Cortex.

[B9] Houde JF, Nagarajan SS, Heinks-Maldonado T (2007). Dynamic cortical imaging of speech compensation for auditory feedback perturbations. Proceedings of the 153rd Meeting of the Acoustical Society of America: Salt Lake City, UT.

[B10] Kalman RE (1960). A new approach to linear filtering and prediction problems. Trans ASME – Journal of Basic Engineering.

[B11] Wolpert DM, Ghahramani Z (2000). Computational principles of movement neuroscience. Nat Neurosci.

[B12] Wolpert DM, Ghahramani Z, Jordan MI (1995). An internal modelfor sensorimotor integration. Science.

[B13] Eulitz C, Diesch E, Pantev C, Hampson S, Elbert T (1995). Magnetic and electric brain activity evoked by the processing of tone and vowel stimuli. J Neurosci.

[B14] Gootjes L, Raij T, Salmelin R, Hari R (1999). Left-hemisphere dominance for processing of vowels: a whole-scalp neuromagnetic study. NeuroReport.

[B15] Heinks-Maldonado TH, Mathalon DH, Houde JF, Gray M, Faustman WO, Ford JM (2007). Relationship of imprecise corollary discharge in Schizophrenia to auditory hallucinations. Arch Gen Psychiatry.

[B16] Saltuklaroglu T, Kalinowski J, Dayalu VN, Stuart A, Rastatter MP (2004). Voluntary stuttering suppresses true stuttering: a window on the speech perception-production link. Perception & Psychophysics.

[B17] Brown S, Ingham RJ, Ingham JC, Laird AR, Fox PT (2005). Stuttered and fluent speech production: an ALE meta-analysis of functional neuroimaging studies. Human Brain Mapping.

[B18] Sun J, Wu J, Li S, Wu Y, Liu L (2003). Localization of the human language cortex by magnetic source imaging. Chinese Medical Journal.

[B19] Biermann-Ruben K, Salmelin R, Schnitzler A (2005). Right rolandic activation during speech perception in stutterers: a MEG study. NeuroImage.

[B20] Bardouille T, Picton TW, Ross B (2006). Correlates of eye blinking as determined by synthetic aperture magnetometry. Clinical Neurophysiology.

[B21] Oshino S, Kato A, Wakayama A, Taniguchi M, Hirata M, Yoshimine T (2007). Magnetoencephalographic analysis of cortical oscillatory activity in patients with brain tumors: synthetic aperture magnetometry (SAM) functional imaging of delta band activity. NeuroImage.

[B22] Sekihara K, Nagarajan SS, Poeppel D, Marantz A (2004). Performance of an MEG adaptive-beamformer source reconstruction technique in the presence of additive low-rank interference. IEEE Trans Biomed Eng.

